# The Mobility of Neurofilaments in Mature Myelinated Axons of Adult Mice

**DOI:** 10.1523/ENEURO.0029-23.2023

**Published:** 2023-03-21

**Authors:** J. Daniel Fenn, Yinyun Li, Jean-Pierre Julien, Peter Jung, Anthony Brown

**Affiliations:** 1 Department of Neuroscience, The Ohio State University, Columbus, Ohio 43210; 2 Medical Scientist Training Program, The Ohio State University, Columbus, Ohio 43210; 3 Quantitative Biology Institute and Department of Physics and Astronomy, Ohio University, Athens, Ohio 45701; 4 CERVO Brain Research Centre, Department of Psychiatry and Neuroscience, Laval University, Quebec, Quebec G1J 2G3, Canada

**Keywords:** neurofilament, axon, axonal transport, photoactivation

## Abstract

Studies in cultured neurons have shown that neurofilaments are cargoes of axonal transport that move rapidly but intermittently along microtubule tracks. However, the extent to which axonal neurofilaments move *in vivo* has been controversial. Some researchers have proposed that most axonally transported neurofilaments are deposited into a persistently stationary network and that only a small proportion of axonal neurofilaments are transported in mature axons. Here we use the fluorescence photoactivation pulse-escape technique to test this hypothesis in intact peripheral nerves of adult male *hThy1-paGFP-NFM* mice, which express low levels of mouse neurofilament protein M tagged with photoactivatable GFP. Neurofilaments were photoactivated in short segments of large, myelinated axons, and the mobility of these fluorescently tagged polymers was determined by analyzing the kinetics of their departure. Our results show that >80% of the fluorescence departed the window within 3 h after activation, indicating a highly mobile neurofilament population. The movement was blocked by glycolytic inhibitors, confirming that it was an active transport process. Thus, we find no evidence for a substantial stationary neurofilament population. By extrapolation of the decay kinetics, we predict that 99% of the neurofilaments would have exited the activation window after 10 h. These data support a dynamic view of the neuronal cytoskeleton in which neurofilaments cycle repeatedly between moving and pausing states throughout their journey along the axon, even in mature myelinated axons. The filaments spend a large proportion of their time pausing, but on a timescale of hours, most of them move.

## Significance Statement

Neurofilaments are space-filling structural polymers that support the growth of axon caliber, which is an important determinant of axonal conduction velocity. Neurofilaments are also cargoes of axonal transport, but the extent to which they move in adult axons has been controversial. The present study resolves this controversy by demonstrating that most, if not all, neurofilaments are mobile in mature myelinated axons of adult mice. Thus, the neurofilament network in axons is remarkably dynamic. This has important implications for the mechanisms by which neurofilament transport and axon morphology are regulated in health and disease.

## Introduction

Neurofilaments, which are the intermediate filaments of nerve cells, are long, flexible cytoskeletal polymers that accumulate in large numbers in myelinated axons during postnatal development. The principal known function of neurofilaments is as space-filling structures that expand axon caliber, which is one important determinant of conduction velocity (Waxman, 1980; Hoffman, 1995). In large axons, neurofilaments are the most abundant structures and occupy most of the axonal volume (Friede and Samorajski, 1970). In electron micrographs, these polymers are aligned in parallel with the long axis of the axon and are spaced apart by side-arm projections that are composed of the C-terminal tail domains of the high-molecular weight neurofilament subunit proteins (Hirokawa et al., 1984; Hisanaga and Hirokawa, 1988; Brown and Hoh, 1997; Mukhopadhyay et al., 2004; Laser-Azogui et al., 2015).

Radioisotopic pulse-labeling studies have shown that neurofilament proteins are synthesized in the soma and transported along axons in the slowest component of axonal transport at average rates of 0.2–8 mm/d (0.002–0.09 μm/s; Hoffman and Lasek, 1975; Lasek et al., 1984). Live-cell imaging of fluorescently tagged neurofilament proteins in cultured nerve cells has shown that the proteins move as assembled neurofilament polymers (Roy et al., 2000; Wang et al., 2000; Wang and Brown, 2001). The filaments move in a stop-and-go manner, alternating between short bouts of rapid movement and pauses of varying duration (Fenn et al., 2018). The movement is bidirectional but with a net anterograde bias (Boyer et al., 2022).

To analyze the pausing behavior of axonal neurofilaments, we developed a fluorescence pulse-labeling technique in which photoactivation is used to mark a population of neurofilaments and the loss of fluorescence caused by neurofilament transport out of the photoactivated region is assayed over time by time-lapse imaging (Trivedi et al., 2007). Since the filaments move rapidly, the fluorescence decay kinetics are dictated largely by the time spent pausing (Li et al., 2014). In multiple studies, we found that the pulse-escape kinetics are biphasic, consistent with two distinct pausing states, which we term “on track” and “off track” (Trivedi et al., 2007; Alami et al., 2009; Monsma et al., 2014; Walker et al., 2019). Neurofilaments in the on-track state alternate between short bouts of rapid movement and short pauses, and can switch to the off-track state in which they may pause for an hour or more. Using computational modeling, we estimated that the filaments spend ≥98% of their time pausing during their transport along axons, with prolonged off-track pauses dominating the kinetic behavior (Li et al., 2014). Thus, the slow axonal transport of neurofilament proteins is a result of rapid movements of neurofilament polymers interrupted by prolonged pauses (Brown, 2000; Brown et al., 2005; Jung and Brown, 2009).

While the axonal transport of neurofilament polymers is widely accepted, the proportion of axonal neurofilaments that are transported *in vivo* has been the subject of controversy for more than three decades. Based on radioisotopic pulse-labeling studies in mouse optic nerve and live imaging in culture neurons, Nixon and Logvinenko (1986) and Yuan et al. (2009, 2015) have proposed that axonally transported neurofilaments are deposited into a stationary network that turns over very slowly. According to their hypothesis, <10% of axonal neurofilaments *in vivo* are transported in a ‘‘stop-and-go’’ manner in mature axons and >90% are fixed in place for months without movement (Yuan et al., 2009, 2012, 2017). This hypothesis was challenged on technical grounds by Lasek et al. (1992), who argued that all axonal neurofilaments move and that there is no persistent stationary population. More recently, we reanalyzed the data of Nixon and Logvinenko (1986) and Yuan et al. (2009) using computational simulations and came to the same conclusion (Li et al., 2012; Brown and Jung, 2013). In the present study, we take advantage of the *hThy1-paGFP-NFM* transgenic mouse (Walker et al., 2019), which expresses a photoactivatable neurofilament protein in neurons, to address this controversy experimentally.

## Materials and Methods

### Mice

All animal procedures were performed in accordance with a protocol approved by The Ohio State University Institutional Animal Care and Use Committee. Production, characterization, and genotyping of the *hThy1-paGFP-NFM* transgenic mice have been described previously (Walker et al., 2019). These mice express neurofilament protein M tagged with a photoactivatable GFP (paGFP-NFM) in neurons driven by the neuron-specific portion of the human Thy1 promoter. The mice were maintained heterozygous for the transgene on a C57BL/6 background. For ease of colony maintenance, all experiments were performed on male mice. In a recent study on neurofilament transport kinetics in tibial nerves from *hThy1-paGFP-NFM* mice, we found no significant difference between male and female mice (Boyer et al., 2022). Mice ranging from 25 to 40 weeks of age were used for the adult mouse pulse-escape experiments, whereas mice ranging from 7 to 16 d of age were used for the juvenile mouse pulse-escape experiments.

### Tibial nerve dissection

Mice were killed by carbon dioxide inhalation followed by cervical dislocation. The tibial nerve was exposed beneath the gastrocnemius muscle using standard dissection techniques (Boyer et al., 2020). The nerve was cut at the knee, where it branches from the sciatic. The proximal end was grasped with fine forceps, and the nerve was lifted gently away from the surrounding tissue, taking care to avoid undue stretching. A second cut was then made distally just above the ankle and the resulting nerve segment (∼2 cm long) was immersed in oxygenated saline solution at room temperature. This saline solution consisted of 98 mm NaCl, 2 mm KH_2_PO_4_, 1 mm KCl, 1 mm MgSO_4_, 1.5 mm CaCl_2_, and 5.6 mm d-glucose, and was oxygenated by bubbling carbogen gas (95%O_2_/5%CO_2_) through the solution for 30 min before use (Breuer et al., 1987; Padilla et al., 1992). Using a binocular dissecting microscope, the epineurium and associated connective tissues were removed gently to allow the superficial axons to sit as close as possible to the glass coverslip. To accomplish this, we took advantage of the fact that the epineurium tends to shrink away from the cut end elastically, exposing the axonal bundle. We grasped the exposed bundle with one pair of fine forceps and the epineurium with another, then peeled the epineurium back in one fluid motion, as though removing a stocking from a leg. This step is important for obtaining the best image quality but must be performed gently because excessive stretching or manipulation of the nerve during the desheathing can result in axonal death.

### Live imaging

All images were acquired using a spinning disk confocal microscope (model Revolution WD, Andor), which included an inverted microscope stand (model TiE, Nikon), a confocal scanning unit (model CSU-W1, Yokogawa), a 100×/1.4 numerical aperture Plan-Apochromat VC oil-immersion objective (Nikon) and an EMCCD (electron-multiplying charge-coupled device) camera (model iXon Ultra 897, Andor) controlled by MetaMorph software (Molecular Devices). The nerve was placed in a heated closed-bath imaging and perfusion chamber (model FCS2, Bioptechs) on the microscope stage and maintained at 37°C. A 100 μm gasket was used between the microaqueduct slide (on top) and the coverslip (below). The temperature of the objective was maintained at 37°C using a temperature controller and objective heater (model H301-T, Okolab). Oxygenated saline was perfused through the chamber at 0.264 ml/min throughout the imaging session using a syringe pump (Sage Instruments/Thermo Fisher Scientific). Before entering the chamber, the saline was warmed to 37°C using an inline solution heater (model SH-27B, Warner Instruments).

Nerves were mounted on the microscope and ready for imaging 25–35 min after mouse death. To obtain the best image quality, we imaged axons on the surface of the nerve touching the coverslip. Suitable fields were located within the middle region of the nerve at least 5 mm from the cut ends and focused under bright-field and epifluorescence illumination. Since the fluorescence of the axons was very weak before photoactivation, we used wide-field confocal bypass mode for these observations and then switched back to confocal mode for the subsequent imaging. Myelinated axons could be identified easily by bright-field imaging because of the distinct optical properties of the myelin sheath. To select fields for imaging, we looked for areas where most of the axons were straight, parallel to each other, and in focus across the field. We avoided areas with cellular debris such as remnants of the epineurium.

### Photoactivation

For pulse-escape experiments, a 5-μm-wide rectangular activation region was drawn across the field of view perpendicular to the axons. This allowed us to activate ∼10–15 axons in each field. The paGFP-NFM was activated with the FRAPPA targeted illumination galvo scanner on the Andor system using a 100 mW 405 nm laser line set to 5% power with five pulses of 40 μs each per pixel and laser blanking using an acousto-optic tunable filter (Walker et al., 2019; Boyer et al., 2020). Depending on the size and orientation of the activation region, the total activation time was 20–30 s.

### Pulse-escape experiments

The activated paGFP fluorescence was excited using a 50 mW 488 nm laser line set to 50% power and imaged with confocality. Camera exposure times were 1 s with the camera electron multiplication gain set to 100. For each pulse-escape series, we acquired the following sequence of images: a preactivation image (immediately before activation), a postactivation image (immediately after activation), and then additional images at 1, 2.5, 5, 10, 15, 30, 60, 90, 120, 150, and 180 min postactivation. There was a delayed increase in the fluorescence within the first minute after activation because of recovery from the dark state (Bancaud et al., 2010), so we normalized the fluorescence intensities in each pulse-escape time course to the image acquired 1 min after photoactivation. Focus was maintained using the Perfect Focus System (Nikon) paired with manual adjustment as necessary. Any manual focusing was performed between fluorescence images using bright-field illumination to avoid additional bleaching of the paGFP fluorescence. Since the total experiment time was 3 h, we were only able to image a single field for each nerve explant.

### Photobleaching calibration

To calibrate the kinetics of photobleaching, we activated the fluorescence in the entire field of view of the camera, waited 1 min for the dark-state recovery, and then acquired images with 1 s exposures every 30 s for a total of 10 min. All imaging conditions were identical to those described above for the pulse-escape experiments. To quantify the bleaching, we measured the fluorescence in a 5 μm window in the center of the field for each axon at each time point. The purpose of measuring the fluorescence in the center of a large activated region and acquiring all images within 10 min of activation was to ensure that the loss of fluorescence could be attributed to photobleaching and not to departure of neurofilaments by axonal transport (Boyer et al., 2020). We averaged the data for multiple axons and fitted the average data to an exponential decay function, which was used to calculate the photobleach correction that was applied to each data point in our analyses.

### Metabolic inhibition

Metabolic inhibition studies were performed as described above, except that we substituted d-glucose with 2-deoxy-d-glucose and added 0.5 mm sodium iodoacetate to the solution. 2-Deoxy-d-glucose is a competitive inhibitor of hexokinase (Bertoni, 1981), and iodoacetate is an irreversible inhibitor of glyceraldehyde-3-phosphate dehydrogenase (Schmidt and Dringen, 2009). After bathing the nerve in this solution, we waited 30 min to allow ATP levels to be depleted before imaging. We then performed pulse-escape experiments as described above, except that we stopped at 30 min. Since the health of the preparations declined over time in the presence of these inhibitors, we imaged only one field per nerve.

### Image processing and analysis

All image analysis was performed using the FIJI/ImageJ software suite with standard plugins (Schindelin et al., 2012; Schneider et al., 2012). Some manual alignment of the images within each image stack was required before quantification to correct for stage drift or slight movement of the nerve in the perfusion chamber during the experiment. This was performed using the TrakEM2 FIJI plugin with semitransparent overlays and using natural features of the images as fiduciary marks. Alignment was necessary for ∼80% of the axons analyzed and typically resulted in a shift of 2–8 pixels in the *X* and/or *Y* direction over the course of a single image series. After alignment, rectangular measurement regions were drawn to delineate the activated regions. The edges of the activated regions and axons were determined using the intensity at half-height in linear intensity profiles drawn parallel or perpendicular to the axis of the axon. Integrated intensity values were then calculated for each time point and corrected for photobleaching using the calibration curve described above. To allow for the dark-state recovery, the corrected intensity measurements were normalized to the intensity at 1 min after activation (see explanation above).

### Data analysis and modeling

Statistical analysis was performed using a two-tailed unpaired *t* test. The Shapiro–Wilk test was used to test for normality, and the *F* test was used to test for equality of the variances. Sample sizes and exact *p* values are reported in the figure legends. Nonlinear curve fitting was performed using the data analysis tools in GraphPad Prism (GraphPad Software). Double-exponential curve fits were constrained to positive values (≥0) but were otherwise unconstrained. The decay function had the following form:

(1) $$y=Ae^{-at}+Be^{-bt}+C\text{,}$$

where A/(A+B+C) and B/(A+B+C) signify the fractions of the filaments in the rapidly and slowly decaying phases at *t* = 0, a and b are the corresponding rate constants, and C/(A+B+C) is the fraction of the filaments in a hypothetical stationary state. If a proportion of the neurofilament population is stationary, then the decay curve is expected to decay to a plateau value given by C.

Rate constants governing the transitions between the moving and pausing states in this model were extracted from the pulse-escape kinetics using a mathematical method described by Li et al. (2014) and used previously by Monsma et al. (2014) and Walker et al. (2019). Specifically, we constructed the average decay curve $y_{a}(t)$ by averaging the remaining fluorescence of all axons y(t) at each time point after activation and fitting the resulting average values to the function above. We then determined the optimal parameters A,B,a,b, and C with a least-square fit. Note that the average value of C was on the order of $10^{-14}$, so any contribution to the extracted rate constants was negligible. We then normalized the curve $y_{a}(t)\to y_{a}(t)/(A+B+C)$ such that $y_{a}\text{(0) = 1}$, then extracted the initial slope [ −(aA+bB)/(A+B+C)] and the long-term decay constant b. These two observables allowed us to express the rate constants $\gamma_{\text{on}}$ and $\gamma_{\text{off }}$ in terms of $\gamma_{01}$ and $\gamma_{10}$. Following the procedure explained in Li et al. (2014), we then determined the values of $\gamma_{01}$ and $\gamma_{10}$ that yielded the closest match to the overall decay in terms of least-squares distance to the experimental data. With those rate constants in place, we used the expressions derived by Li et al. (2014) to calculate specific parameters of neurofilament transport, including the average proportion of the time neurofilaments spend on-track and off-track, the average duration of the on-track and off-track pauses, and the average proportion of the time spent moving. The net average velocity was estimated using the expression $\bar{v}=p_{a}v_{a}+p_{r}v_{r}$ (Boyer et al., 2022), where $p_{a}$ and $p_{r}$ are the fractions of all neurofilaments in the anterograde and retrograde moving states measured in mouse tibial nerve by Boyer et al. (2022), and $v_{a}$ and $v_{r}$ are taken to be 0.5 μm/s and -0.5 μm/s, respectively, based on our measurements in cultured neurons (Li et al., 2014).

## Results

### Imaging tibial nerve *ex vivo*

**Figure 1.**
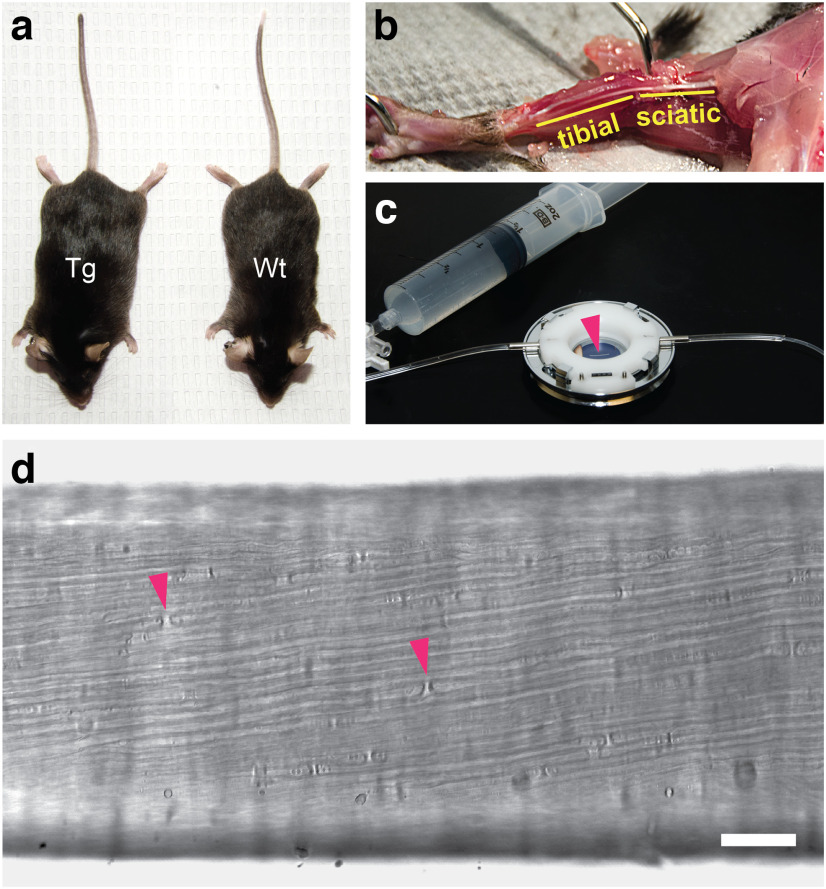
Tibial nerve dissection and imaging. ***a***, Wild-type (Wt) and heterozygous *hThy1-paGFP-NFM* transgenic (Tg) male sibling mice at 24 weeks of age. ***b***, Dissection exposes the tibial nerve, which branches from the sciatic nerve at the knee and extends to the foot. ***c***, A heated stage-top imaging and perfusion chamber was used to keep the nerve segment (arrowhead) at physiological temperature. The chamber was perfused with oxygenated saline using a syringe pump. ***d***, A low-power image of the tibial nerve as seen by bright-field microscopy. Myelinated axons can be detected because of the refractility of the myelin sheaths. Arrowheads point to two nodes of Ranvier. Scale bar, 0.1 mm.

Tibial nerves were dissected from adult heterozygous *hThy1-paGFP-NFM* transgenic mice and mounted in a heated imaging and perfusion chamber with a constant flow of oxygenated saline (Fig. 1). The cut ends of the axons constrict and reseal by a Ca^++^ and SNARE-dependent vesicular fusion repair mechanism (Bloom and Morgan, 2011; Spaeth et al., 2012), and imaging of organelle movement in such nerve preparations has shown that the axons remain viable for at least 3 h (Viancour and Kreiter, 1993; Walker et al., 2019).

**Figure 2.**
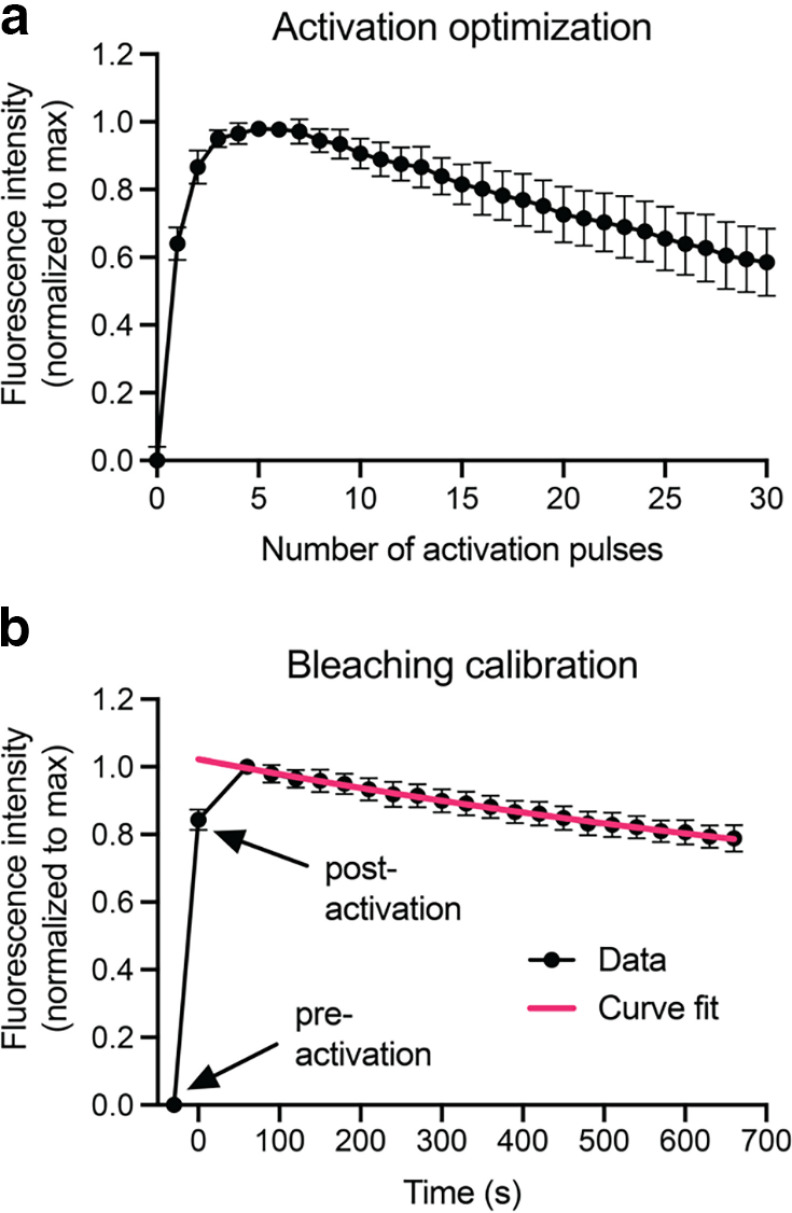
paGFP-NFM activation and bleaching. ***a***, Optimization of paGFP-NFM activation using consecutive 40 μs pulses of 405 nm laser light (14 axons from 3 activation fields). Maximal activation was achieved with 5 pulses. ***b***, Calibration of the photobleaching of paGFP-NFM excited with a 488 nm laser light (46 axons from 6 activation fields). Note that the fluorescence increases during the first minute after activation because of delayed recovery from the dark state (see text), so we incorporated a 1 min delay into all our pulse-escape experiments and normalized the fluorescence at later times to the fluorescence intensity at 1 min. The exponential curve fit represents the photobleaching calibration curve used in this study. The fluorescence declined by 13% after 11 exposures, which is the number of exposures we used for our pulse-escape experiments below. The error bars represent the SD.

We have shown previously that *hThy1-paGFP-NFM* mice express paGFP-NFM in all axons of the sciatic and tibial nerves and that the transgene expression level is 1.6% of the expression level of endogenous NFM (Walker et al., 2019). To photoactivate the paGFP-NFM fluorescence, we used a galvo scanner to illuminate a selected area of the nerve with violet (405 nm) laser light. To optimize the activation time, we fixed the laser power at 5% and the pulse duration at 40 μs, and then exposed the axons to 30 pulses of light, acquiring an image of the green fluorescence using 488 nm excitation after each pulse. The fluorescence intensity peaked after five pulses and then declined with additional pulses, so we selected five pulses for all subsequent experiments (Fig. 2*a*). The decline in fluorescence with additional pulses occurs because, in addition to activating the paGFP, violet light also excites the activated protein leading to photobleaching of the green fluorescence.

To correct for photobleaching during image acquisition, we used a photobleaching correction curve. This was created by activating paGFP across an entire field of axons and measuring the fluorescent decay in a 5 μm window (Fig. 2*b*). The fluorescence increased by ∼15% within the first minute after photoactivation because of relaxation from the dark state (Bancaud et al., 2010) and then declined exponentially with each successive exposure because of photobleaching. We used the expression for this exponential decay to correct the experimental data below (see Materials and Methods).

### Photoactivation of axons from young and adult mice

**Figure 3.**
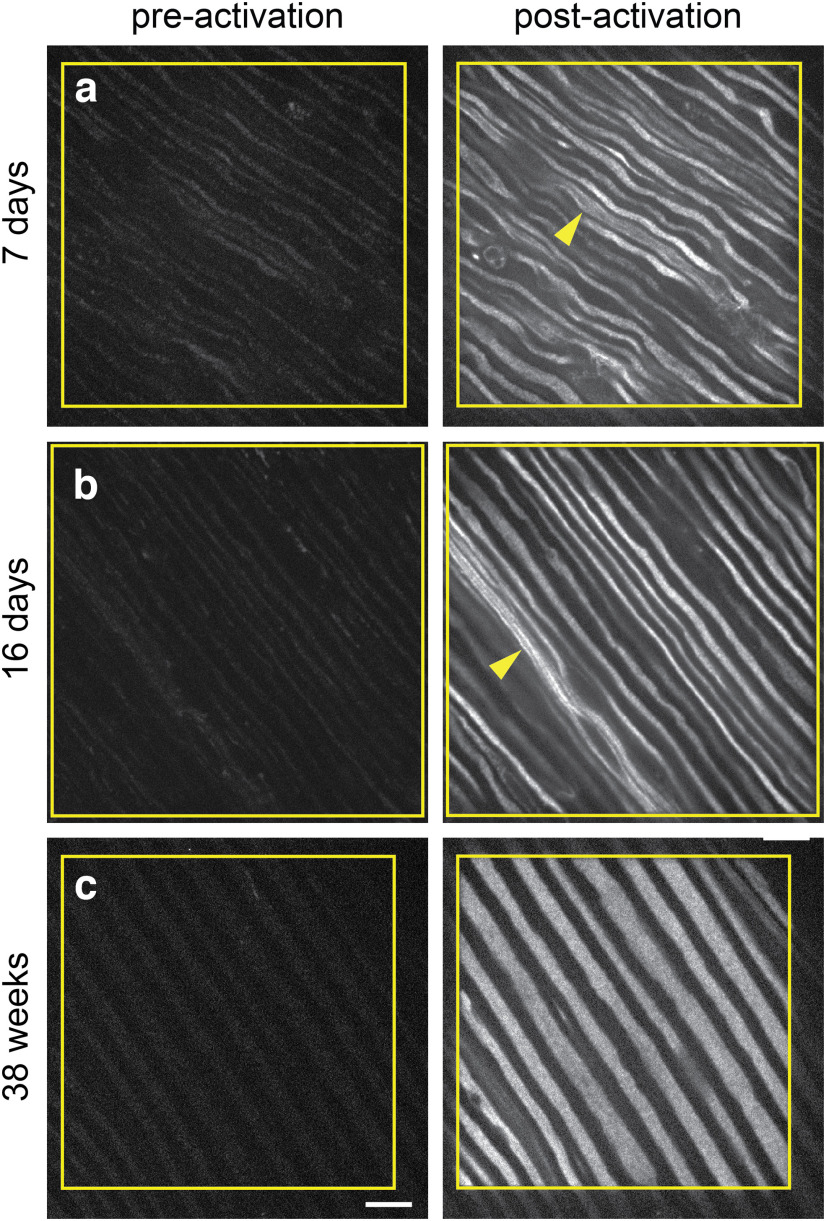
paGFP-NFM is expressed throughout postnatal development and into adulthood. ***a–c***, Whole-field activation of paGFP-NFM in nerves from *hThy1-paGFP-NFM* mice aged 7 d (***a***), 16 d (***b***), and 36 weeks (***c***). The yellow boxes show the area of the nerve that was activated. Individual axons can be seen running diagonally across the field. The arrows denote what appear to be bundles of unmyelinated axons (Remak bundles) in the younger nerves. Note the expansion of axon caliber with age, which is driven by neurofilament accumulation. Scale bar, 10 μm.

The neuron-specific portion of the human Thy1 promoter has been reported to drive the expression of transgenes in mice starting at birth (Morris, 1985; Kelley et al., 1994; Caroni, 1997). Expression increases rapidly in the first postnatal week and then continues to increase, reaching a stable level at ∼4 weeks that is maintained into adulthood (Kollias et al., 1987). To confirm this, we photoactivated tibial nerves from mice ranging from 1 to 40 weeks. We observed photoactivation of all myelinated axons throughout the nerves at all ages within this range, confirming early and sustained expression of the paGFP-NFM in these mice (Fig. 3).

### Pulse-escape experiments

**Figure 4.**
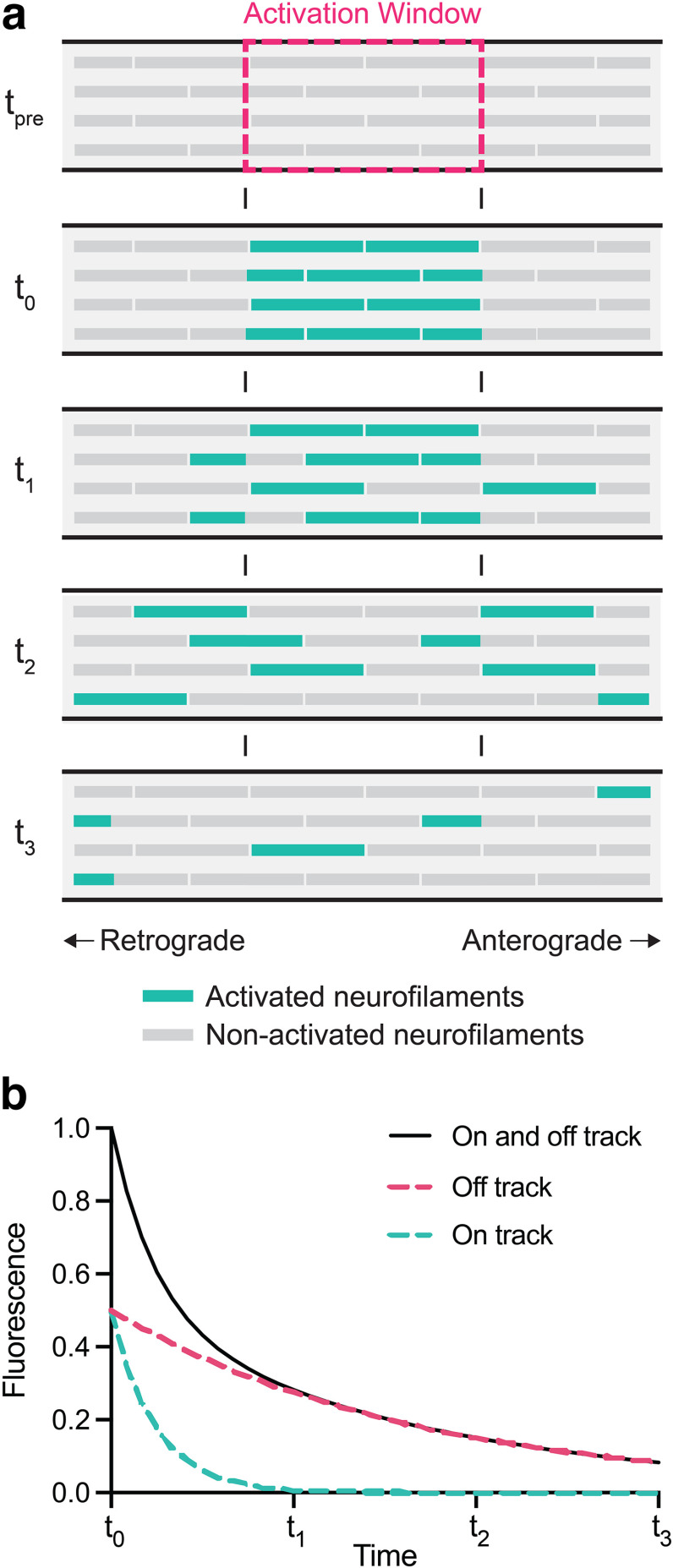
The pulse-escape method. ***a***, Schematic diagram of a pulse-escape experiment in one axon. Neurofilaments are activated (teal) in a short segment of an axon and then gradually depart the activated region (dashed magenta box) because of their rapid intermittent movement. ***b***, Measurement of the fluorescence intensity in the activation window. The decay kinetics (black) are biphasic and can thus be analyzed into two components. The initial rapidly declining phase is caused by the departure of neurofilaments in the “on-track” state (teal), which move more frequently, superimposed on a slower departure of neurofilaments in the “off-track” state (magenta).

To test the hypothesis that there is a large and persistently stationary population of neurofilaments in mature axons, we analyzed the neurofilament transport kinetics using the fluorescence photoactivation pulse-escape technique, which permits analysis of the long-term pausing behavior of neurofilaments (Trivedi et al., 2007; Li et al., 2014). This method involves photoactivation of the neurofilaments in a short segment of an axon and then analysis of the fluorescence decay. Neurofilaments depart the activated region because of their bidirectional transport, resulting in a decline in the fluorescence intensity with time (Fig. 4). We have shown previously that the kinetics in this experimental paradigm are biphasic, with an initial rapidly declining phase and a later more slowly declining phase. This is consistent across multiple studies and preparations including myelinated and unmyelinated axons in cell culture, as well as myelinated axons *ex vivo* (Trivedi et al., 2007; Alami et al., 2009; Monsma et al., 2014; Walker et al., 2019). Since neurofilaments move at micrometers per second and the activated region measures just 5 μm across, the kinetics of departure are dictated largely by the pause durations. Thus, the biphasic kinetics indicate distinct pausing states for neurofilaments, which we term on-track and off-track (Trivedi et al., 2007; Li et al., 2014). On-track filaments engage in bouts of rapid movement interrupted by short pauses (average duration on the order of minutes), whereas off-track filaments pause for prolonged periods (average duration on the order of ≥1 h). The initial rapidly declining phase represents filaments that happened to be in the on-track state at the time of activation and therefore departed more rapidly. Once these filaments have departed, the decay kinetics transition to a slower phase that reflects the mobilization of off-track filaments into the on-track state (Fig. 4*b*).

To quantify the loss of fluorescence from the activated regions, we performed pulse-escape imaging on 48 axons from 10 different animals ranging in age from 25 to 40 weeks. Since we have shown previously that the axons can be maintained on the microscope stage for 3 h with perfusion of oxygenated saline (Walker et al., 2019), we set this as the duration of our pulse-escape experiments and performed one pulse-escape experiment per nerve, with each nerve coming from a different animal. Each field of view on the microscope contained 10–15 myelinated axons. As the axons are predominantly parallel, we routinely activated all the axons at one time using a rectangular region 5 μm in width drawn perpendicularly to the axons across the entire field. For analysis, we selected internodes with uniform morphology that were in focus across the entire field (typically three to five axons met these criteria per field). Axons were rejected if they did not remain in focus for the entire time course or if they exhibited unusual morphologic features such as myelin figures. The activated axons ranged from 1.6 to 5.3 μm in diameter with an average diameter of 3.1 μm. Based on the cross-sectional neurofilament density in the mouse sciatic nerve (175/μm^2^), this corresponds to ∼350–3860 neurofilaments per axonal cross-section (Reles and Friede, 1991).

**Figure 5.**
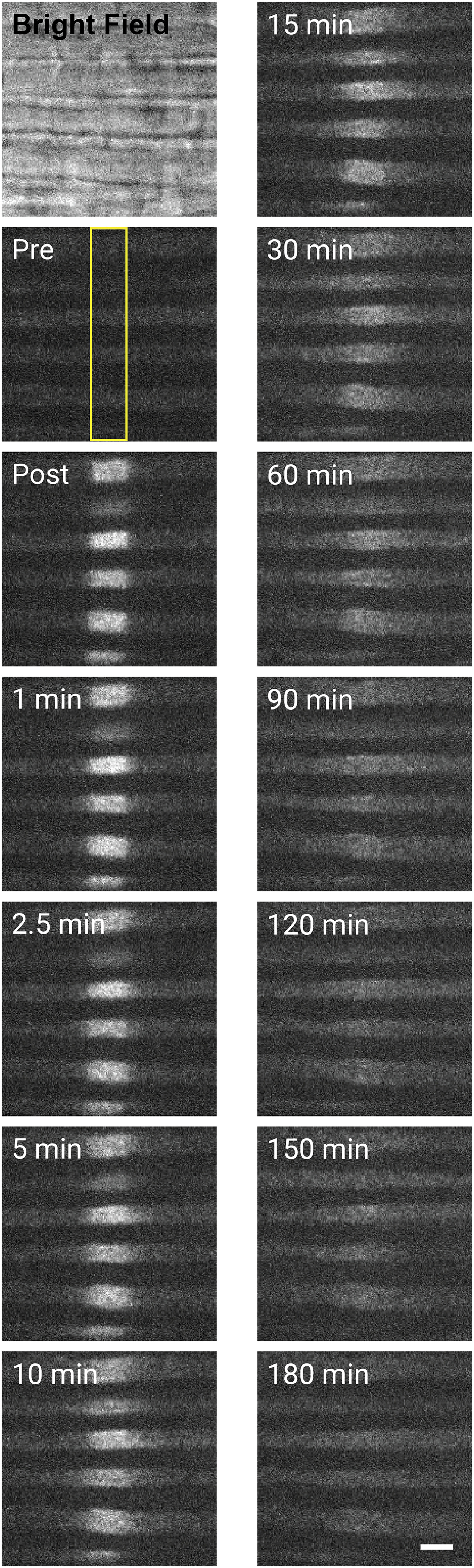
A pulse-escape experiment. The bright-field image shows the myelin sheaths surrounding each individual axon. The axons are visible faintly in the preactivation image (Pre). The yellow box marks the activation window. Immediately after photoactivation (Post), a 5 μm-long segment of each axon is fluorescent. The bidirectional transport of the fluorescent filaments results in a dispersal of the filaments that is evident by a blurring of the proximal and distal ends of the activated region at early times leading to a gradual smearing out of the fluorescence in both directions at later times. Little fluorescence remains in the activated region after 180 min. Scale bar, 5 μm.

Figure 5 shows a series of images from a pulse-escape experiment, and Movie 1 is a representative movie. The axons are spaced apart by their myelin sheaths, which can be detected in the bright-field image because of their distinct optical properties. The axons are weakly fluorescent before photoactivation because of autofluorescence and weak excitation of the unconverted paGFP with blue light. Illumination with violet light results in a substantial increase in the excitation of the protein with blue light, resulting in a substantial increase in the green fluorescence (Patterson and Lippincott-Schwartz, 2002). The boundaries of the photoactivated region are sharp immediately after photoactivation and then blur both proximally and distally with time, reflecting the bidirectional movement of the photoactivated polymers out of the activated region. The low expression level of the paGFP-NFM in these axons is not sufficient to permit the detection of single-neurofilament polymers, but the activated fluorescence is visible because of the large number of neurofilaments per cross section (Boyer et al., 2020). The activated region blurs progressively as the filaments disperse in both directions along the axon. Little fluorescence remains in the original location after 180 min.

**Figure 6.**
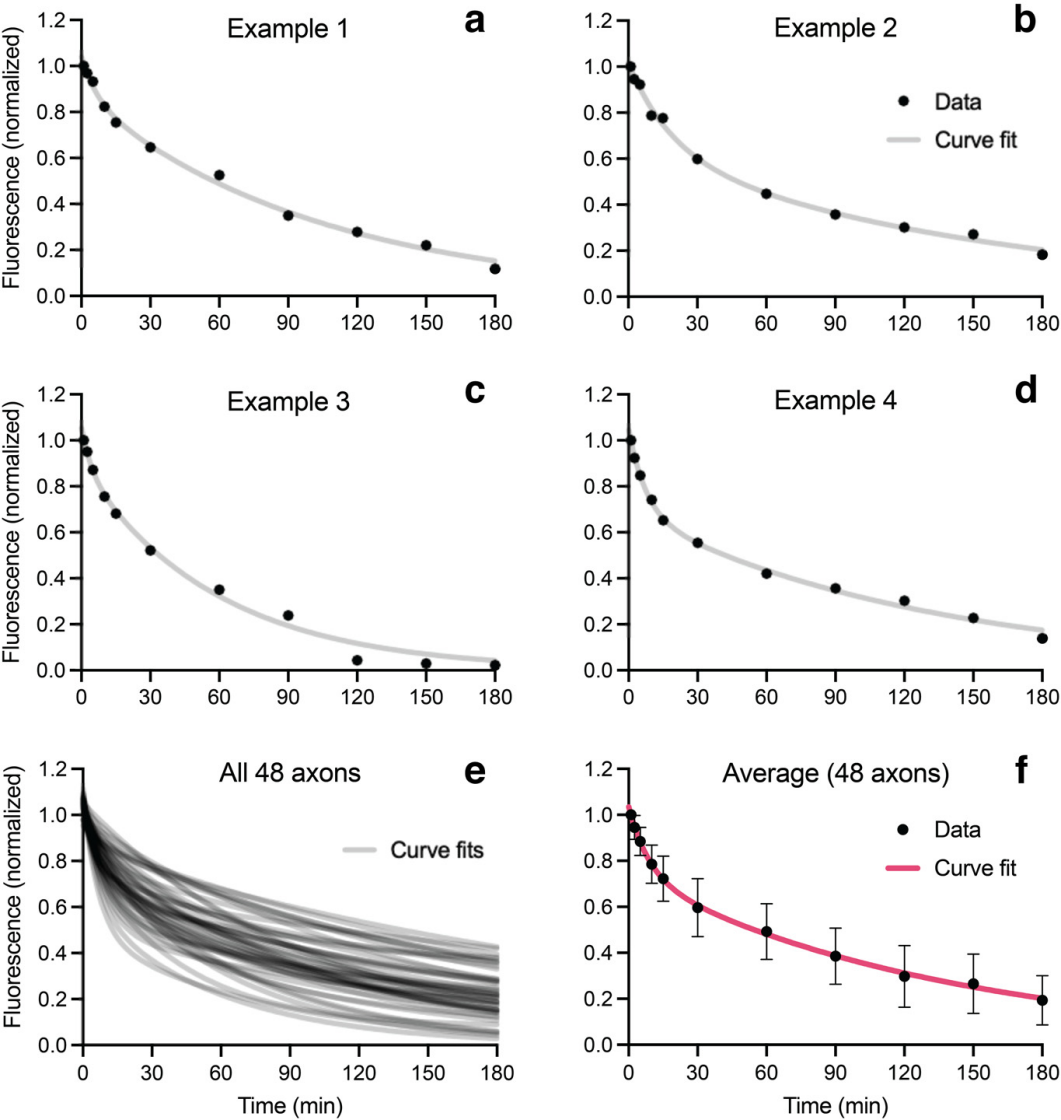
Pulse-escape kinetics. ***a–d***, Examples of the pulse-escape kinetics for 4 different axons. The curve fits are a least-squares fit of the data to a double-exponential function. ***e***, Double-exponential curve fits for 48 axons from 10 animals. ***f***, Average of the data for all 48 axons, showing the double-exponential curve fit to the average data (magenta line). All data were corrected for photobleaching as described in the Methods. The error bars represent the SD. After 3 h, ∼81% of fluorescence had departed the activated region (average percentage remaining = 19 ± 11%).

**Figure 7.**
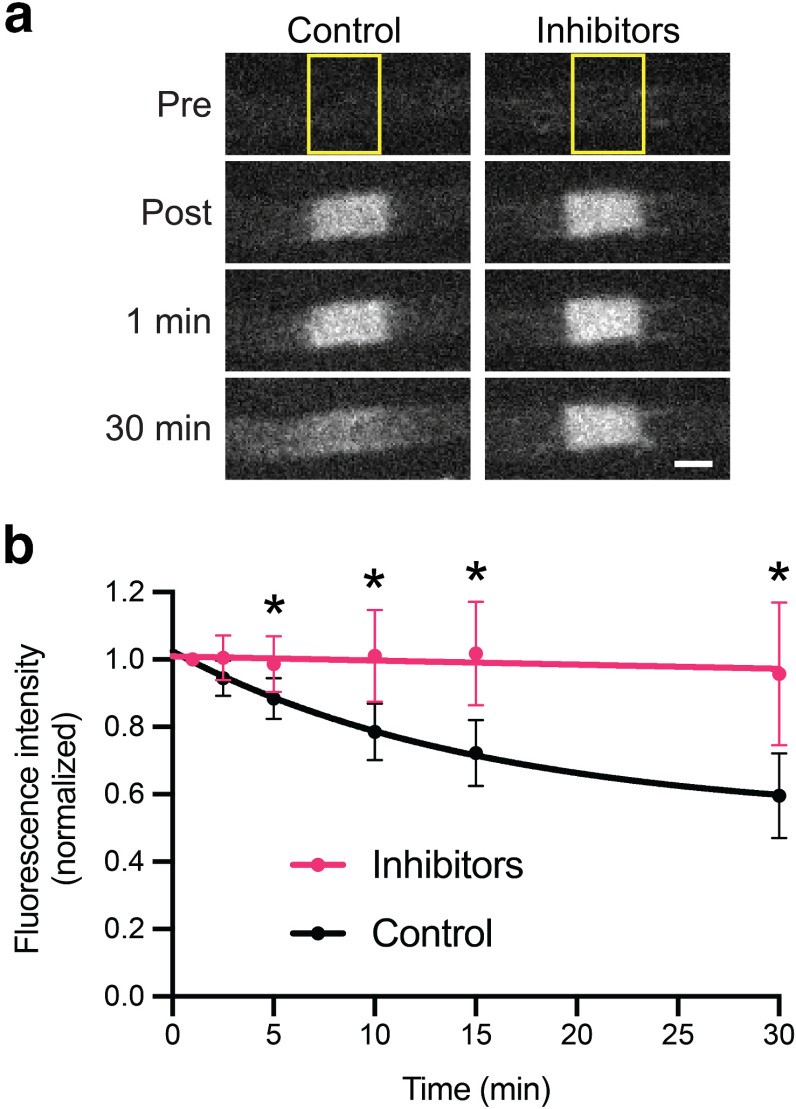
Neurofilament transport is blocked by glycolytic inhibitors. Nerves were immersed for 30 min in saline containing 5.6 mm d-glucose (control) or saline containing 5.6 mm 2-deoxy-d-glucose plus 0.5 mm sodium iodoacetate (inhibitors) to inhibit glycolysis. ***a***, Examples of single myelinated axons imaged before photoactivation (Pre), immediately after photoactivation (Post), and then 1 min and 30 min later. Note that the fluorescence disperses both proximally and distally in the control because of the bidirectional transport of the filaments, but not in the presence of inhibitors. The yellow boxes mark the activated regions. Scale bar, 3 μm. ***b***, Quantification of the pulse-escape kinetics for axons in saline (48 axons from 10 animals; data from Fig. 6) or saline plus inhibitors (25 axons from 6 animals). All data were corrected for photobleaching as described in the Methods. The error bars represent the SD. The asterisks denote a statistically significant difference (*t* = 5 min, *p *=* *7 × 10^−8^; *t* = 10 min, 1 × 10^−8^; *t* = 15 min, 3 × 10^−10^; *t* = 30 min, 5 × 10^−9^; two-tailed unpaired *t* test). The curves are least-squares fits of the data to an exponential function.

Figure 6 shows examples of the pulse-escape kinetics for 4 axons plus the curve fits and the average of all 48 axons. All axons exhibited the same characteristic biphasic decay, but there was considerable variation in the slopes of the two phases and the transition between them. On average, 72% of the fluorescent neurofilaments remained after 15 min, declining to 60% after 30 min and 48% after 60 min. By 3 h, 19% of the fluorescence remained, meaning that 81% of the photoactivated neurofilaments had departed the activated region. Treatment of the nerves with glycolytic inhibitors blocked the loss of fluorescence, confirming that the movement is an active transport process with negligible contribution of diffusion (Fig. 7, Movie 2).

Movie 1. A pulse-escape experiment. The movie consists of the following sequence of images: a preactivation image (immediately before activation), a postactivation image (immediately after activation), and then additional images at 1, 2.5, 5, 10, 15, 30, 60, 90, 120, 150, and 180 min postactivation. The field of view contains six myelinated axons, four of which are fully in the focal plane. The length of the activated regions is 5 μm. Note that the bidirectional transport of the fluorescent filaments is manifested as a blurring of the proximal and distal ends of the activated regions at early times leading to a gradual smearing out of the fluorescence in both directions at later times. 10.1523/ENEURO.0029-23.2023.video.1

Movie 2. Neurofilament transport is blocked by treatment with saline containing 5.6 mm 2-deoxy-d-glucose and 0.5 mm sodium iodoacetate to inhibit glycolysis. The movie consists of the following sequence of images: a preactivation image (immediately before activation), a postactivation image (immediately after activation), and then additional images at 1, 2.5, 5, 10, 15, and 30 min postactivation. The field of view contains five myelinated axons, three of which are fully in the focal plane. The length of the activated regions is 5 μm. Note that the activated regions remain sharp and had negligible loss of fluorescence over the course of the experiment. The autofluorescent structures that appear during the movie are mitochondria, which exhibit enhanced flavin autofluorescence under metabolic stress (Huang et al., 2002; Surre et al., 2018; Boyer et al., 2020). 10.1523/ENEURO.0029-23.2023.video.2

### Extrapolation to longer times

**Figure 8.**
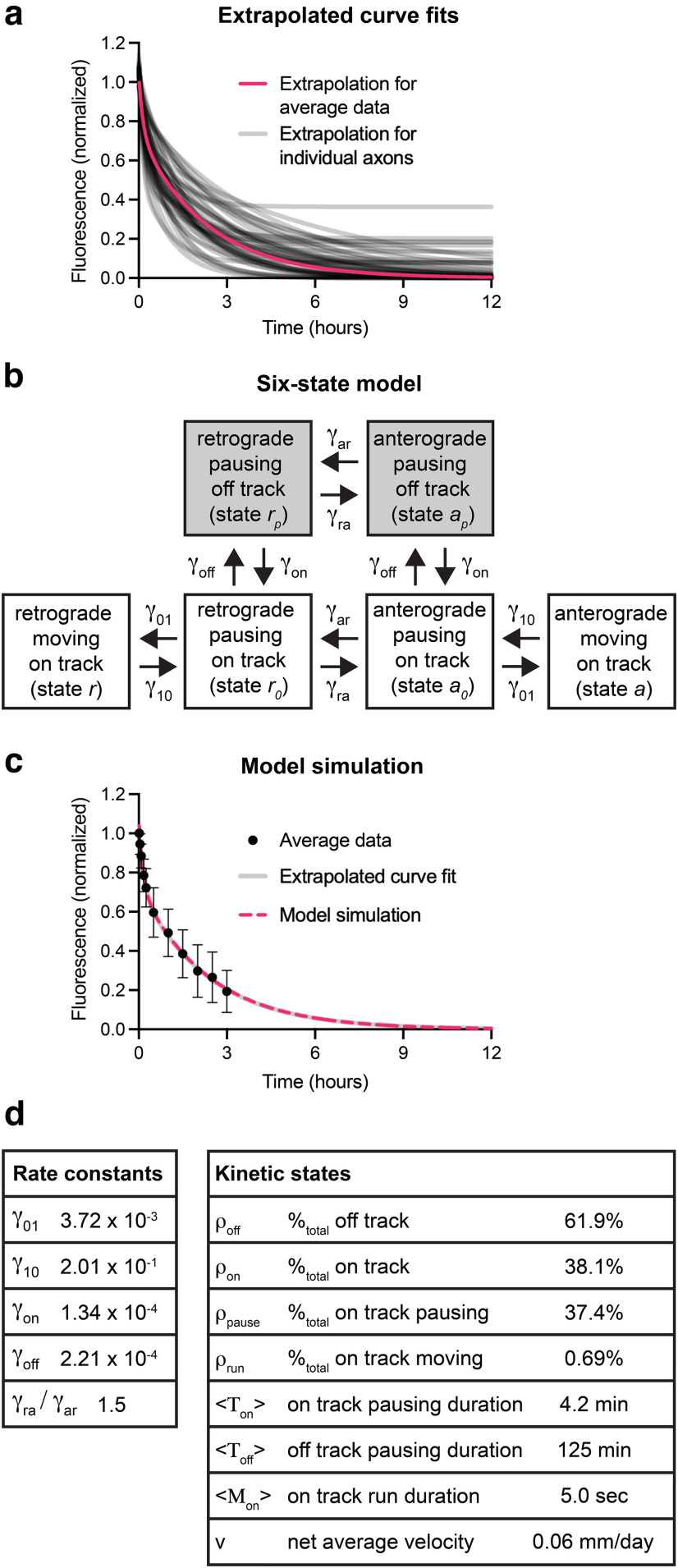
Mathematical modeling of the neurofilament transport kinetics. ***a***, Double-exponential curve fits for all 48 axons individually (gray lines) or for the average data (magenta line), extrapolated to 12 h after photoactivation. ***b***, Schematic of the six-state model of neurofilament transport (Jung and Brown, 2009). Neurofilaments can be either on-track or off-track. On-track neurofilaments move rapidly and intermittently in both anterograde and retrograde directions, switching between moving states ( a and r) and short-term pausing states ( $a_{0}$ and $r_{0}$). While pausing on-track, they can move off-track and enter a long-term pausing state ( $a_{p}$ and $r_{p}$). Transitions between the on-track moving and pausing states are given by the rate constants $\gamma_{01}$ and $\gamma_{10}$. Transitions between the on-track and off-track pausing states are given by $\gamma_{\text{on}}$ and $\gamma_{\text{off}}$. Neurofilaments in both the on-track and off-track pausing states can reverse direction, governed by the rate constants $\gamma_{ar}$ and $\gamma_{ra}$. ***c***, Simulated pulse-escape experiment (dashed magenta line) overlaid on a double-exponential curve fit to the average data (gray line). ***d***, Table of the rate constants extracted from the model and the kinetic parameters predicted by those rate constants. The equations relating the rate constants to the kinetic parameters were derived from the study by Li et al. (2014). The ratio of the reversal rate constants $\gamma_{ra}$/ $\gamma_{ar}$ was obtained using a modification of the pulse-escape technique, which we call “pulse-spread” (Boyer et al., 2022).

In the above experiments, we were limited to a 3 h imaging window because of the viability of the nerve preparation *ex vivo*. After 3 h, only 19% of the fluorescent neurofilaments remained in the activated regions, but the fluorescence appeared to be continuing to decline. Since the decay kinetics follow a double-exponential decay, we performed a least-squares regression fit of the data for each axon to this function and extrapolated to longer times. To allow for the possibility of a hypothetical stationary component, we added the “plateau” parameter C (Eq.1) and did not constrain the function to decay to zero (see Materials and Methods). Figure 8*a* shows the resulting extrapolated curve fits (gray lines) as well as a curve fit to the average data (magenta line). All but 1 of the 48 axons was predicted to plateau at <20% of the original fluorescence, and the average plateau value was 4.4% (range, 0–36%; *n* = 48). Extrapolating the curve fit to the average data, the fluorescence was predicted to decay essentially to zero, with 1% remaining after 10 h. Thus, the decay kinetics in the first 3 h demonstrate that on average any stationary neurofilament population in these axons is <19% of the total population, and extrapolation predicts that on average 99% of the filaments would be expected to have left the activated region after 10 h.

### Computational simulation of the stop-and-go model

The biphasic decay kinetics observed in our experimental data are predicted by our six-state model of neurofilament transport (Fig. 8*b*). Mathematical analysis of the decay kinetics in terms of this model has revealed that the initial slope at short times and exponential decay at long times have different dependencies on the rate constants of the model, allowing us to constrain the model sufficiently to obtain an optimal match to the data (Li et al., 2014; see Materials and Methods). We have also previously derived mathematical expressions that relate key parameters of neurofilament transport to the rate constants in the model (Li et al., 2014).

Figure 8*c* shows a simulated pulse-escape experiment (dashed magenta line) obtained using rate constants optimized to match the experimental data and superimposed on the extrapolated double-exponential curve fit (gray line). Figure 8*d* shows the rate constants and the calculated kinetic parameters. The on-track rate constants $\gamma_{01}$ and $\gamma_{10}$ were $\text{3}{\text{.72 × 10}}^{–\text{3}}{\text{ s}}^{–\text{1}}$ and $\text{2}{\text{.01 × 10}}^{–\text{1}}{\text{ s}}^{–\text{1}}$, respectively. Off-track filaments moved on-track at a rate of ${\text{γ}}_{\text{on}}\text{ = 1}{\text{.34 × 10}}^{–\text{4}}{\text{ s}}^{–\text{1}}$, while on-track filaments moved off-track at a rate of ${\text{γ}}_{\text{off}}\text{ = 2}{\text{.21 × 10}}^{–\text{4}}{\text{ s}}^{–\text{1}}$. These values are within the same order of magnitude as previously reported values for myelinated internodes in mouse tibial nerve (Walker et al., 2019).

Figure 8*d* shows various parameters of neurofilament transport calculated from the optimized rate constants. We found that the filaments spent an average of 62% of the time in the off-track state with average pause times of 125 min and 38% in the on-track state with average pause times of 4.2 min. Considering both on-track and off-track pauses, the filaments spent 0.7% of their time moving with an average bout duration of 5 s. To estimate the net average velocity, we need to know the relative proportion of the time that the filaments spent moving in the anterograde and retrograde directions and the velocity of the bouts of movement (see Materials and Methods). For the bout velocities, we use *v_a_* = 0.5 μm/s and *v_r_* = –0.5 μm/s based on our measurements obtained in cultured neurons (Li et al., 2014). The reversal rate constants cannot be obtained from the pulse-escape kinetics because the method is blind to the direction in which the filaments depart the activated region (Li et al., 2014). Thus, to estimate the average velocity, which is dependent on the balance of anterograde and retrograde movements, we use our recent measurements in mouse tibial nerves (60% anterograde, 40% retrograde) obtained using a related approach called the pulse-spread method (Boyer et al., 2022). Based on these assumptions, we obtain a net average neurofilament velocity of 0.06 mm/d. This is approximately twofold slower than measurements in the distal mouse sciatic nerve (0.12 mm/d) obtained using radioisotopic pulse labeling, which is consistent with reports that neurofilament transport slows with distance along axons (Hoffman et al., 1985b; Watson et al., 1989; Xu and Tung, 2001). The slow velocity reflects the fact that the filaments spend 99.3% of their time pausing. However, over a period of hours, the data indicate that most filaments move.

## Discussion

**Figure 9.**
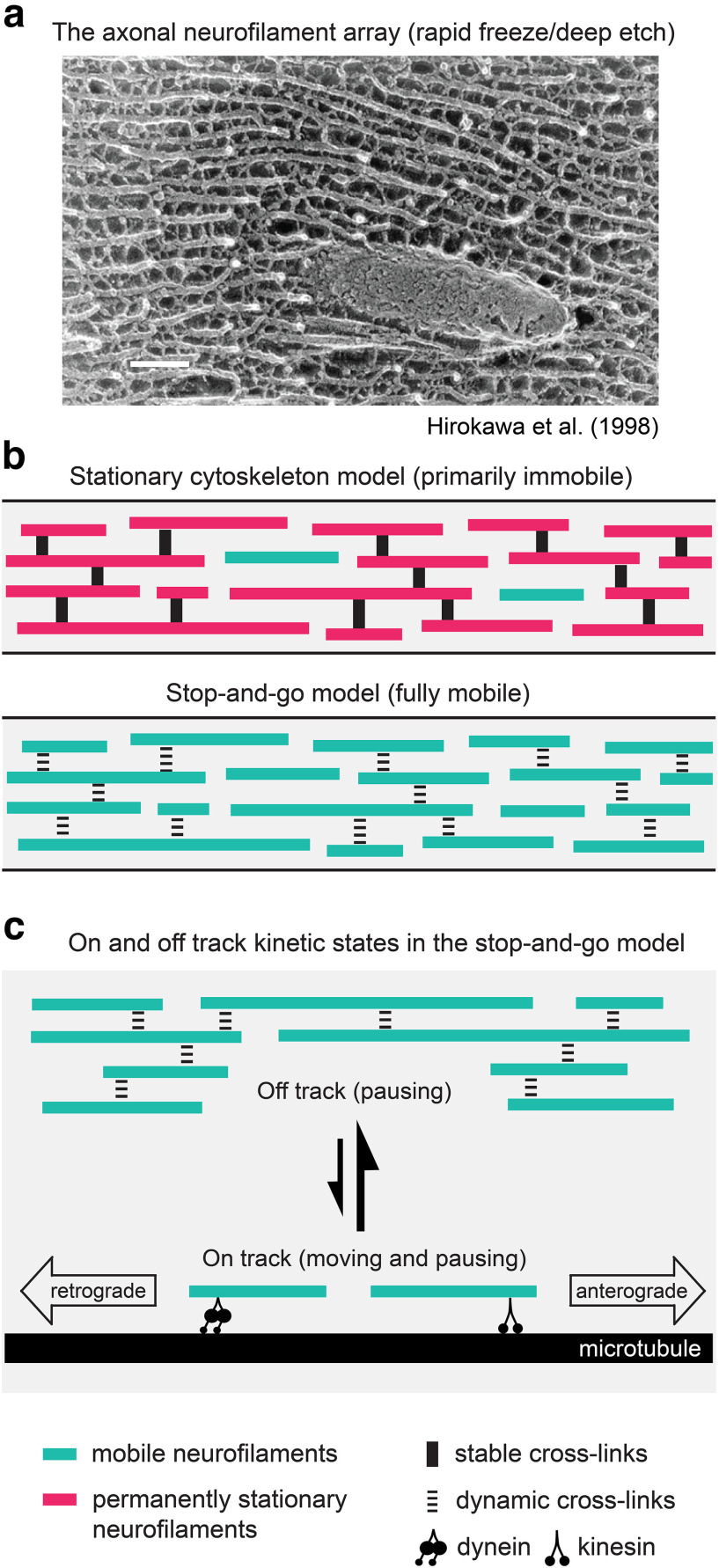
The static model versus the dynamic model. ***a***, Image of the axonal cytoskeleton obtained by rapid-freeze/deep-etch electron microscopy showing an extensive array of neurofilaments interconnected by their side-arm projections. Reproduced from the article by Hirokawa and Takeda (1998). Scale bar, 100 nm. ***b***, The stationary cytoskeleton model proposes that the cross-links seen in ***a*** create a stationary neurofilament network (magenta) and that only a small proportion of the neurofilaments (teal) are transported. In contrast, the stop-and-go model considers that all neurofilaments are intermittently mobile, implying that the cross-links are weak and/or dynamic. ***c***, In the stop-and-go model, neurofilaments cycle between an off-track state, in which they may pause for ≥1 h, and an on-track state, in which they move rapidly and intermittently, alternating between short bouts of rapid movement and pauses lasting for seconds or minutes. While the filaments spend >99% of their time pausing, over a period of multiple hours all neurofilaments move.

Though controversial, the hypothesis that axonal neurofilaments are deposited into a stationary neurofilament network has persisted for over 3 decades and has had a profound influence on how researchers think about neurofilament organization and dynamics (Barry et al., 2007; Millecamps et al., 2007; Sunil et al., 2012; LoPachin and Gavin, 2015; Yuan et al., 2017). We believe that the durability of this hypothesis is due partly to electron microscopic images, which give the visual impression that axonal neurofilaments form an extensively cross-linked network. Particularly influential in this regard were the rapid-freeze, deep-etch platinum replica images obtained by Hirokawa (1982) and Hirokawa et al. (1984) in which neighboring filaments appear to be extensively interconnected by their radial sidearm projections (Fig. 9*a*).

In the present study, we used *hThy1-paGFP-NFM* transgenic mice to test for the existence of a stationary neurofilament network in mature myelinated axons. After photoactivation of neurofilaments in short segments of these axons, ∼80% departed the activated region within 3 h. This movement was blocked by the inhibition of glycolysis, confirming that it was an active transport mechanism that cannot be attributed to diffusion or disassembly of the neurofilament polymers. We were not able to extend the treatment with glycolytic inhibitors for >30 min, so technically we cannot exclude the possibility that there was some contribution of neurofilament disassembly to the loss of fluorescence after that time. However, there is no precedent for such disassembly in healthy axons, and it is highly unlikely given the known stability of neurofilament polymers. Thus, we conclude conservatively that at least 80% of the neurofilaments in these axons are mobile on a timescale of 3 h and that any stationary population cannot exceed 20% of the total neurofilament population. These data are not consistent with the stationary neurofilament hypothesis, which proposes that most neurofilaments are deposited into a persistently stationary cytoskeleton that remains fixed in place for months (Yuan et al., 2009, 2017). Using computational modeling, we estimated that the filaments spent >99% of their time pausing. However, the average off-track pause time was ∼2 h, and extrapolation of the decay kinetics beyond 3 h predicted that essentially all of the filaments would leave the activated region within half a day.

A possible criticism of our study is that it was performed in excised nerves and not in living mice. While the axons reseal and are viable for at least 3 h in oxygenated saline, the dissection is nonetheless an injury. Formally speaking, it is possible that neurofilament mobility might be increased in response to this injury. However, studies on slow axonal transport *in vivo* using radioisotopic pulse-labeling have shown that, while there is an increase in the velocity of neurofilament transport during axon regeneration, injury itself induces no such increase (Hoffman and Lasek, 1980; McKerracher and Hirscheimer, 1992). Thus, there is no reason to believe that the mobility of the neurofilaments in our study is an artifact of nerve excision.

A key assumption in our experiments is that the fluorescent neurofilament protein incorporates throughout the axonal neurofilament array and is thus representative of the entire axonal neurofilament population. We believe this is valid because the hThy1 promoter becomes active in the first postnatal week, which is early in the period of axonal maturation during which the stationary cytoskeleton is hypothesized to be deposited. For example, the axonal cross-sectional area in mouse peripheral nerves increases from ∼3.1 μm^2^ at 7 d to ∼17.8 μm^2^ at 90 d (Sherman et al., 2012). Based on published neurofilament densities in mouse peripheral nerves (Friede and Samorajski, 1970), this corresponds to an increase from an average of ∼100 filaments per axon at 7 d to ∼685 filaments per axon at 90 d. Thus, >85% of the neurofilaments in the axons at day 90 must have entered those axons after day 7. Our experiments were performed in mice 25–40 weeks of age, which is >6 months after the initiation of *hThy1-paGFP-NFM* expression.

Yuan et al. (2009) used the pulse-escape method to analyze the mobility of neurofilaments in primary cultures of cortical neurons. Instead of using photoactivation, these authors used a conventional fluorescent fusion protein and created a pseudoactivated region by photobleaching the flanking regions. Using this approach, they reported that neurofilament mobility decreased with increasing neurofilament content. Thus, Yuan et al. (2009) proposed that the formation of the stationary neurofilament network requires a certain critical mass of neurofilaments, presumably reached in mature, adult neurons. They noted that the rapid intermittent movement of neurofilaments in axons can only be observed directly in cultured neurons that have a relatively low neurofilament content because this is the only way to resolve single neurofilaments (Wang et al., 2000; Wang and Brown, 2001; Fenn et al., 2018). Thus, they proposed that the extensive movement of neurofilaments reported in cultured neurons is a feature of immature axons and is not representative of the neurofilament dynamics in mature axons *in vivo*.

**Figure 10.**
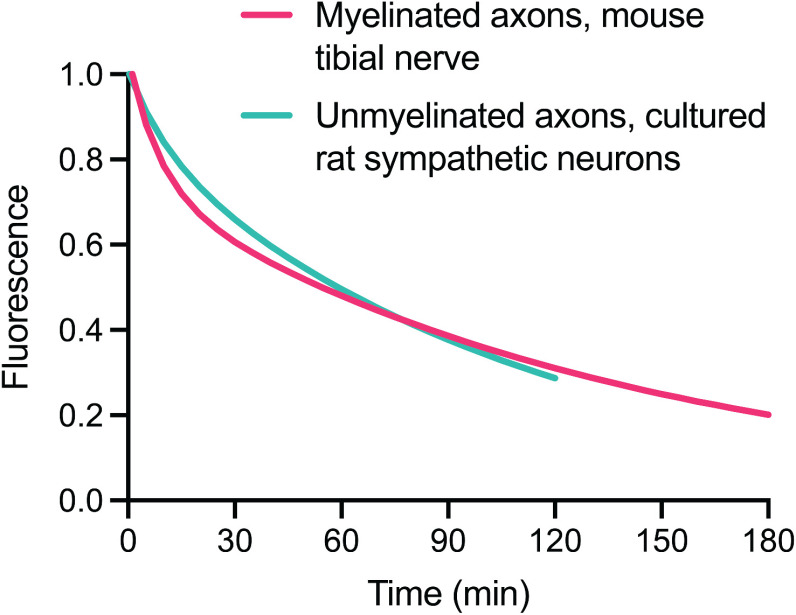
Neurofilament mobility in cultured nerve cells and *in vivo.* Comparison of the average pulse-escape kinetics in myelinated axons of mouse tibial nerves (magenta, present study) with our previously published data obtained in unmyelinated axons of sympathetic neurons in primary cell culture (teal; Trivedi et al., 2007). Note the similarity in the decay kinetics.

To explore this, we compared the pulse-escape kinetics that we obtained in myelinated axons of adult mouse tibial nerves with previously published data for unmyelinated axons of sympathetic neurons cultured from neonatal rat superior cervical ganglia (Trivedi et al., 2007; Fig. 10). The unmyelinated axons in culture contain just a few neurofilaments per axonal cross section, whereas myelinated axons in adult mice contain hundreds or thousands of neurofilaments per cross section (Friede and Samorajski, 1970). However, the decay kinetics are remarkably similar, with ∼70% of the filaments leaving the activated region within 2 h in both cases. The average time spent pausing was estimated to be ∼97% for the unmyelinated axons in culture, with average pause durations of 29 s on-track and 61 min off-track (Trivedi et al., 2007). This compares to an average time spent pausing of 99.3% for the myelinated axons in the present study, with average pause durations of 4.2 min on-track and 125 min off-track. Thus, while our analysis does predict that neurofilaments spend more time pausing in myelinated axons *in vivo*, the similarity in the decay kinetics underscores the remarkably dynamic nature of the neurofilament network *in vivo* and demonstrates that the mobility of axonal neurofilaments is not unique to immature axons.

Our conclusions on the mobility of axonal neurofilaments are consistent with the results of radioisotopic pulse-labeling studies in laboratory animals. For example, we have shown using computational modeling that the kinetics of neurofilament transport in mouse sciatic and optic nerves can be explained by a stop-and-go-model in which all neurofilaments move at a broad range of rates (Brown et al., 2005; Jung and Brown, 2009; Li et al., 2012). In mouse optic nerves, our simulations predicted that the average neurofilament cycled on-track and off-track >200 times over a period of 10 d, with average on-track and off-track pause durations of ∼20 s and 60 min, respectively (Li et al., 2012). Using the analytical solutions described in the study by Jung and Brown (2009), we calculated that on average the filaments in the present study spent 99.3% of their time pausing, but the probability of a neurofilament remaining paused off-track for >12 h was <0.001%. In other words, the filaments pause a lot, but they are mobile on a time course of hours.

The mobility of axonal neurofilaments may seem inconsistent with the images of extensively cross-linked neurofilament networks obtained by electron microscopy (Fig. 9*a*). However, strength of interaction cannot be assessed directly based on electron micrographs. In fact, there are multiple lines of evidence to suggest that neurofilaments are only weakly interactive. First, while neurofilaments are spaced regularly in axonal cross-sections when packed densely, at lower densities they are distributed randomly and do not cluster (Price et al., 1988). Second, neurofilaments diffuse apart from each other in osmotically swelled axoplasm, again showing no tendency to cluster (Brown and Lasek, 1993). Third, biophysical experiments using atomic force microscopy indicate that the neurofilament sidearms interact through long-range repulsive forces and function more to space adjacent filaments apart rather than to link them together (Brown and Hoh, 1997; Kumar and Hoh, 2004; Mukhopadhyay et al., 2004; Stevens and Hoh, 2010). The charge distribution on the sidearms is predicted to result in short-range “handshake” electrostatic interactions between sidearms from neighboring filaments, but the strength of these interactions is on the order of the thermal energy of the molecular environment and thus highly labile (Beck et al., 2010). In this regard, it is important to note that the images showing cross-bridges between neurofilaments (Fig. 9*a*) were obtained by chemically fixing the tissue before freezing (e.g. Hirokawa, 1982; Hirokawa et al., 1984). Such cross-links are much less apparent in the images of Schnapp and Reese (1982), who froze the tissue without prior chemical fixation. Therefore, the apparent cross-links observed by electron microscopy may represent weak and/or transient interactions that are stabilized by chemical fixation, creating the impression of a network that appears far more static and interconnected in micrographs than it is.

In conclusion, the experimental data in this study and in others discussed above favor a dynamic model of the axonal cytoskeleton in which the interactions between neurofilaments are mostly weak or reversible, allowing these cytoskeletal polymers to cycle freely between mobile and immobile states during their journey down the axon (Fig. 9*b*,*c*). The filaments spend most of their time pausing, but the average pause duration is on the order of hours rather than weeks or months. The result is a slow movement with a net anterograde bias that is sufficient to supply neurofilaments to the growing axon during development and continues even in adult myelinated axons that are no longer growing. An obvious question is why does the neuron continue to invest energy to move neurofilaments in axons once axons have reached their mature length and caliber? We believe that the most likely answer is that a mobile neurofilament population is the simplest way to establish and maintain a uniform axonal morphology. To understand this, it is important to appreciate that axons need to maintain a uniform diameter for long distances. Since neurofilaments occupy most of the axonal volume, this requires a uniform neurofilament distribution. Models in which the filaments are deposited into a static phase during their transport along the axon cannot easily explain this uniform distribution. For example, we have shown previously that a simple deposition model based on a fixed deposition rate generates a highly nonuniform neurofilament distribution characterized by a proximal-to-distal gradient of declining neurofilament content (Li et al., 2012). To avoid this, it is necessary to assume a spatially modulated deposition rate that increases with distance along the nerve and decreases with time during postnatal development, reaching zero when the axon attains its mature caliber. While this is possible, it requires complex spatial and temporal regulation. In contrast, a model in which neurofilaments exhibit a net anterograde movement throughout the life of the axon naturally establishes and maintains a uniform neurofilament distribution and axonal morphology without the need to invoke such complex regulation (Nowier et al., 2023).

A potentially important consequence of the persistent mobility of axonal neurofilaments is that perturbations of neurofilament transport would be expected to result in a redistribution of these polymers, even in mature adult axons. Thus, a mobile neurofilament cytoskeleton may explain the dramatic accumulation or depletion of axonal neurofilaments that is observed in mature adult axons in disease (De Vos et al., 2008; Millecamps and Julien, 2013). A mobile neurofilament cytoskeleton can also explain the transient reduction in axonal neurofilament content that occurs during axon regeneration after injury (Hoffman et al., 1985a; Nowier et al., 2023). Such changes are hard to reconcile with a largely static cytoskeleton.

## Synthesis

Reviewing Editor: Eran Perlson, Tel Aviv University Decisions are customarily a result of the Reviewing Editor and the peer reviewers coming together and discussing their recommendations until a consensus is reached. When revisions are invited, a fact-based synthesis statement explaining their decision and outlining what is needed to prepare a revision will be listed below. The following reviewer(s) agreed to reveal their identity: NONE. Note: If this manuscript was transferred from JNeurosci and a decision was made to accept the manuscript without peer review, a brief statement to this effect will instead be what is listed below.

The manuscript by Fenn et al, is well written and provides compelling evidence that a large proportion of neurofilaments in the tibial nerve of adult rodents are mobile. The neurofilament transport literature would otherwise forever include the idea of a permanently stationary population of neurofilaments in the axon. This work resolves the issue satisfactorily for the first time in decades and thereby corrects this point of view which would otherwise have persisted in the literature. The explanations in all parts of the manuscript are excellent and detailed. A computational modeling component fortifies the experimental results. Well done schematics are helpful.

The authors, however, should consider clarifying two issues either by referring to prior studies or discussing the ramifications of the current experiments.

1) Could the ex vivo nerve preparation impact neurofilament motility? Nerves are injured during the removal - could injury provide signals that promote increased motility? This is not easy to address, but the possibility should be discussed. If the model is transferable to an in vivo situation, why would it be advantageous for virtually all NFs to be motile rather than static in the intact uninjured adult axon?

2) Glycolysis inhibitors block the loss of fluorescence at the activation site, supporting the idea that active transport and not diffusion is responsible. However, can slow disassembly of NFs by enzymes be ruled out? If kinesin or dynein inhibitors (or MT depolymerizing agents) prevented loss of fluorescence it would add to the evidence that MT-based transport is important.

## Author Response

Response to reviewers

We thank the editor and reviewers for their comments, which have helped us to improve our discussion. We have uploaded a clean version of the revised manuscript as a Word document as well as a version with all our changes highlighted.

CRITIQUE:

1) “Could the ex vivo nerve preparation impact neurofilament motility? Nerves are injured during the removal - could injury provide signals that promote increased motility? This is not easy to address, but the possibility should be discussed.”

AUTHOR RESPONSE:

While the axons reseal and are viable for at least three hours in oxygenated saline, the reviewers are correct that the dissection is nonetheless an injury. Formally speaking, it is possible that stationary neurofilaments might be mobilized in response to injury. However, studies on slow axonal transport in vivo using radioisotopic pulse-labeling have shown that, while there is an increase in the velocity of neurofilament transport in regenerating axons, injury itself induces no increase (Hoffman & Lasek, 1980; McKerracher et al, 1992) Thus, it is unlikely that the neurofilament mobility observed in our study was induced by excision of the nerve from the animal. We have added a paragraph addressing this in the Discussion (see paragraph 3 of the revised Discussion).

CRITIQUE:

“If the model is transferable to an in vivo situation, why would it be advantageous for virtually all NFs to be motile rather than static in the intact uninjured adult axon?”

AUTHOR RESPONSE:

We thank the reviewers for raising this question. While we can only speculate about the possible advantages, we agree that it is important to do so. We believe the most likely explanation is that a mobile neurofilament population is the simplest way to establish and maintain a uniform axonal morphology. To understand this, it is important to appreciate that axons need to maintain a uniform diameter for long distances. Since neurofilaments occupy most of the axonal volume, this requires a uniform neurofilament distribution. Models in which the filaments are deposited into a static phase during their transport along the axon cannot easily explain this uniform distribution. For example, we have shown previously that a simple deposition model based on a fixed deposition rate generates a highly non-uniform neurofilament distribution characterized by a proximal-to-distal gradient of declining neurofilament content (Li et al, 2012). To avoid this, it is necessary to assume a spatially modulated deposition rate that increases with distance along the nerve and decreases with time during post-natal development, reaching zero when the axon attains its mature caliber. While not impossible, this requires complex spatial and temporal regulation. In contrast, a model in which neurofilaments exhibit a net anterograde movement along the axon throughout the life of the axon can readily establish and maintain a uniform neurofilament distribution and axonal morphology without the need to invoke such complex regulation (Nowier et al, 2023). We also note that the mobility of axonal neurofilaments can explain the dramatic accumulation or depletion of axonal neurofilaments that is observed in disease and in response to injury even in mature adult axons. This again is something that is hard to explain if the cytoskeleton is static. We have revised the last paragraph of the Discussion to make these ideas more explicit.

CRITIQUE:

2) “Glycolysis inhibitors block the loss of fluorescence at the activation site, supporting the idea that active transport and not diffusion is responsible. However, can slow disassembly of NFs by enzymes be ruled out? If kinesin or dynein inhibitors (or MT depolymerizing agents) prevented loss of fluorescence it would add to the evidence that MT-based transport is important.”

AUTHOR RESPONSE:

The fact that we observed no measurable loss of fluorescence over 30 minutes in the presence of glycolytic inhibitors argues against any contribution of either diffusion or disassembly to the pulse-escape kinetics. While we cannot disprove the possibility that the neurofilaments started to disassemble slowly after 30 minutes, there is no precedent for neurofilament disassembly in healthy axons and it is highly unlikely given the known stability of neurofilament polymers. We have addressed this in paragraph 2 of the Discussion. We have not attempted to inhibit microtubule motors or induce microtubule disassembly in these nerve preparations. The former would require the identification of inhibitors with suitable specificity and efficacy and the ability to permeate myelinated axons. The latter would likely be challenging because of the existence of highly stable microtubules in mature axons. We are not aware of the use of microtubule-disassembling drugs on mature myelinated axons, but even in cultured neurons it can take many hours to achieve full microtubule disassembly in axons (e.g. with nocodazole).

ADDITIONAL MINOR EDITS:

1. p.4 - Added a sentence to clarify that neurofilament movement is bidirectional

2. Fig. 1 - altered intensity scaling and orientation in panel d and labeling in panels a and d to improve esthetics and layout

3. Fig. 6 - corrected the line color in panel C

4. Fig. 7 - deleted a stray asterisk in the figure

5. Added some new references to the bibliography

6. Updated the reference to Nowier et al to “in press”
